# An evaluation of factors associated with taking and responding positive to the tuberculin skin test in individuals with HIV/AIDS

**DOI:** 10.1186/1471-2458-11-687

**Published:** 2011-09-05

**Authors:** Líbia CRV Moura, Ricardo AA Ximenes, Heloísa L Ramos, Demócrito B Miranda Filho, Carolina DP Freitas, Rosangela MS Silva, Isabella Coimbra, Joanna d'Arc L Batista, Ulisses R Montarroyos, Maria de Fátima P Militão Albuquerque

**Affiliations:** 1 Department of Tropical Medicine, Universidade Federal de Pernambuco, Recife, Brazil; 2 Department of Medical Science, Universidade de Pernambuco, Recife, Brazil; 3 NESC Department, Centro de Pesquisas Aggeu Magalhães/FIOCRUZ, Recife, Brazil

## Abstract

**Background:**

The tuberculin skin test (TST) is still the standard test for detecting latent infection by *M tuberculosis *(LTBI). Given that the Brazilian Health Ministry recommends that the treatment of latent tuberculosis (LTBI) should be guided by the TST results, the present study sets out to describe the coverage of administering the TST in people living with HIV at two referral health centers in the city of Recife, where TST is offered to all patients. In addition, factors associated with the non-application of the test and with positive TST results were also analyzed.

**Methods:**

A cross-sectional study was carried out with HIV patients, aged 18 years or over, attending outpatient clinics at the Correia Picanço Hospital/SES/PE and the Oswaldo Cruz/UPE University Hospital, who had been recommended to take the TST, in the period between November 2007 and February 2010. Univariate and multivariate logistic regression analyses were carried out to establish associations between the dependent variable - taking the TST (yes/no), at a first stage analysis, and the independent variables, followed by a second stage analysis considering a positive TST as the dependent variable. The odds ratio was calculated as the measure of association and the confidence interval (CI) at 95% as the measure of accuracy of the estimate.

**Results:**

Of the 2,290 patients recruited, 1087 (47.5%) took the TST. Of the 1,087 patients who took the tuberculin skin test, the prevalence of TST ≥ 5 mm was 21.6% among patients with CD4 ≥ 200 and 9.49% among those with CD4 < 200 (p = 0.002). The patients most likely not to take the test were: men, people aged under 39 years, people with low educational levels and crack users. The risk for not taking the TST was statiscally different for health service. Patients who presented better immunity (CD4 ≥ 200) were more than two and a half times more likely to test positive that those with higher levels of immunodeficiency (CD4 < 200).

**Conclusions:**

Considering that the TST is recommended by the Brazilian health authorities, coverage for taking the test was very low. The most serious implication of this is that LTBI treatment was not carried out for the unidentified TST-positive patients, who may consequently go on to develop TB and eventually die.

## Background

The HIV epidemic has had serious consequences around the world and especially in developing countries. One of the gravest of these has been an increase in the incidence of tuberculosis (TB) [1]. In the population not infected with HIV, 5% of those infected with *M. tuberculosis *may develop primary TB within the first two years following exposure and another 5% may develop post-primary TB later in life. By contrast, co-infected individuals stand a 5-10% chance each year of developing TB [2,3].

In recent years, considerable investment has been made available for research into tuberculosis control programs, including the introduction of various strategies to contain the advance of this epidemic [1,4,5]. However, despite the adopted strategies, there has been an increase in the prevalence of HIV, which has resulted in a rise in the incidence of TB [6]. Tuberculosis is still the leading cause of death in HIV positive patients in various parts of the world, especially in countries where the disease is rife and resources are limited [7-9].

Clearly, the risk of developing TB is lower in patients infected with HIV who receive HAART. However, TB still occurs even when patients are undergoing HAART [8,10-13]. In countries with a low prevalence of TB/HIV co-infection the use of HAART is associated with a marked reduction in the incidence of many opportunistic infections [10,14-16], while in countries with a high prevalence of co-infection it is still unclear whether this protective effect occurs [17]. To establish a substantial impact on the incidence of TB, it would be necessary to begin HAART early in the course of HIV infection, with a high level of coverage and adherence [18].

The effectiveness of LTBI treatment with isoniazid in people living with HIV has been reported since the last decade. The majority of clinical trials and prospective cohort studies have shown that treatment with isoniazid for six or 12 month periods prevents tuberculosis and is safe in people infected with HIV and with TST ≥ 5 mm [19-25]. Based on the existing evidence, LTBI treatment with daily doses of isoniazid for six or nine months is recommended in individuals living with HIV [26,27].

HIV positive patients with a reactive TST (≥ 5 mm) have a significantly higher risk of developing full-blown TB than those who do not react (< 5 mm) [28-30]. A systematic review of randomized clinical trials shows that the treatment of LTBI based on a reactive TST reduces the risk of active TB in patients with HIV [31]. Evidence such as this provides grounds for the use of TST and the subsequent treatment of LTBI [32,33].

The tuberculin skin test (TST), based on the detection of a delayed hypersensitivity reaction to *Purified Protein Derivative *(PPD) is still the standard test for detecting infection by *M. tuberculosis*. In order to conduct this test, two visits are needed, one for the inoculation and another to read and interpret the test results [32,33], Although it is a simple test requiring only low-cost materials, with no need for the presence of a laboratory specialist and is extensively used in contexts where resources are limited, epidemiological studies and annual investigations [34,35] have nonetheless shown that there are practical difficulties associated with administering the TST. Coverage differences exist for administering the TST in different health services, suggesting that the motivation of health staff is important when caring for HIV-positive patients [28]. On the other hand, the low sensitivity and specificity of the TST has cast some doubt on its performance and, as a consequence, it has been less requested by attending physicians [12,28]. There is also concern that LTBI treatment using isoniazid alone may lead to resistance to this drug [36,37] as well as potential side-effects [24,38,39]. However, TST is still used worldwide for LTBI screening in individuals with HIV/AIDS, including developing countries [40].

From a clinical perspective, the diagnosis and treatment of LTBI are important measures in reducing the risk of progression to active TB and its associated complications [6]. It is believed, that where an effective logistical system for acquiring and distributing the TST and isoniazid (INH) was established, both physicians and patients would be encouraged to take the TST and read the results [17,26,41].

Given that the Brazilian Health Ministry recommends that LTBI treatment should be guided by the results of the TST [42-44] the present study aims to describe the coverage of administrating the TST in people living with HIV/AIDS, attended at two health centers in the city of Recife, where TST is available. In addition the factors associated with the non-application of the test, and with a positive TST result in patients infected with HIV, were also analyzed.

## Methods

### Study Population, Recruitment, and Survey Methods

A cross-sectional study was carried out with people living with HIV/AIDS, aged 18 years or over, attending outpatient facilities at the Correia Picanço Hospital/SES/PE and the Oswaldo Cruz/UPE University Hospital, which are referral services for HIV/AIDS in the city of Recife, Brazil, between November 2007 and February 2010.

Patients were excluded if they had a previous history of or were undergoing treatment for tuberculosis or LTBI.

Patients were first informed of the purpose of the research and, once they had agreed to participate, were asked to sign the terms of free informed consent. Information was gathered by way of interviews and the application of a questionnaire designed specifically for this study. In addition to the questionnaire, a special form was used by the principal researcher to obtain data from medical records.

The tuberculin skin test was carried out by trained technicians at each health center, using the Mantoux technique. A 0.1 ml dose of PPD (*purified protein derivative*) RT 23, was applied intradermally in the middle third of the left forearm. The test was considered to be reactive when an induration ≥ 5 mm was detected, 72 hours after administering the PPD.

In view of the known difficulty of incorporating this test into clinical practice, the research plan included a lecture for attending physicians, at each of the services covered by the study. The talk was delivered by a specialist in pneumology with extensive experience in tuberculosis, in an attempt to raise awareness among physicians of the importance of performing the TST and treating LTBI, when recommended, in patients with HIV/AIDS. Talks were also arranged for patients at the two centers as a way of informing them of the risk of contracting TB and of the importance of the TST in screening for LTBI.

The study was carried out in two stages. In the first stage, involving all the patients in the cohort making up the study sample, the coverage of TST testing was calculated and factors associated with not taking the test were investigated; in a second stage factors associated with testing positive on the TST were analyzed only for the group of patients who actually took the test.

### Definition of Variables

In the first analysis, the dependent variable was having taken the tuberculin skin test, considering patients who did not take the TST as cases and those who did as the control group.

The independent variables examined were: biological variables (sex and age), socio-demographic factors (place of residence, years of schooling, hospital attended), life habits (alcohol consumption - categorized as abstinent, light drinker, heavy drinker, and people undergoing alcohol dependence treatment -, smoking - categorized as non-smoker, former smoker, and smoker -, marijuana use, cocaine use, and crack use) and clinical variables (history of contact with tuberculosis, body mass index (BMI), use of HAART, time on HAART, presence of AIDS, CD4 T-lymphocyte count)

For the alcohol consumption variable, patients were classified as teetotal if they reported that they never drank or drank less than eight units a year; a light drinker if they drank a maximum of two days a week, without exceeding ten units per month; a heavy drinker if they drank in excess of five doses a day at least 3 to 4 days a week, and as dependent if they were undergoing treatment. For the smoking variable, non-smokers were considered to be those who had never smoked in their life, former smokers those who had quit smoking at least six months before the date of inclusion in the study; and smokers those who were smoking on the day they entered the study or who had quit less than six months before this date.

In the second analysis, the dependent variable relating to the result of the tuberculin skin test was classified on the basis of patients who reacted to the TST (≥ 5 mm) and the controls, which did not react (0-4 mm). The independent variables were the same used for the first stage.

### Data Analysis

For both stages of the study, the χ^2 ^test was used to test the statistical significance of associations and the p value was calculated. A univariate analysis was conducted of the association between the dependent variable and the independent variables, using the odds ratio (OR) as the measure of association (OR) and a confidence interval (CI) of 95% as the measure of accuracy of the estimate. Analysis of multivariate logistic regression used the backward stepwise method for selecting variables, thereby including in the model all variables associated with failure to conduct the tuberculin skin test for the first stage, and with a positive TST for a second stage, with a level of significance of p < 0.25 in the univariate analysis. The final model retained variables whose association with failure to conduct the TST for the first stage, and with a positive TST for a second stage were statistically significant at a level of p ≤ 0.05.

The study was approved by the Ethics Committee at the Federal University of Pernambuco for research involving human beings (SISNEP FR-067159/CAAE-0004.1.172.106-05/REGISTRO CEP/CCS/UFPE 254/05).

## Results

Between November 2007 and February 2010, 2,290 patients were recruited for the study. Of these, 1,425 (62.2%) were male and the mean age of the patients was 39.36 years. Most of the patients included in the study were non-white (73.4%), lived as part of a family (80.8%) and did not have a steady partner (53.5%). 87.8% were literate and 58.6% had up to nine years of schooling. In relation to life habits, 38.3% consumed alcohol, 27.3% used marijuana, 9.2% cocaine, 6.9% crack and 55.1% were smokers.

A total of 641(28%) patients related contact with tuberculosis, while information on close contact with smear positive TB was unavailable.

**Figure 1:**
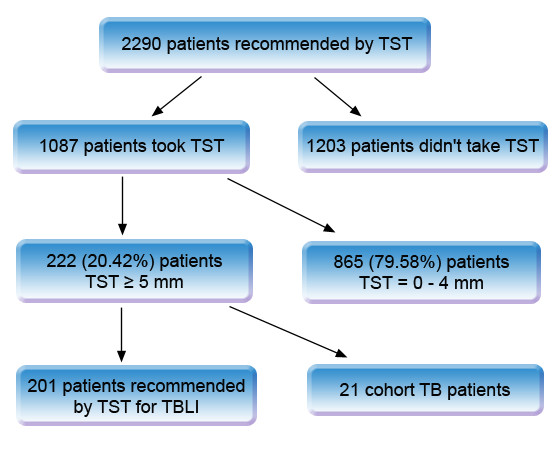
**Flowchart of the study population**.

Of the 2,290 patients, 1087 (47.5%) took the tuberculin skin test (TST) (Figure 1). The mean age of the group who took the TST was 40.4 years (± 0.3) compared with 38.4 years (± 0.3) for the group who did not. The difference between these two means was not statistically significant. Tables 1 and 2 show the percentage distributions for the variables studied, chi square and p value.

**Table 1:** Distribution of biological and socio-economic characteristics of people living with HIV by adherence to TST and TST reactivity, Recife 2007-2010.

VARIABLES	WITH TST		WITHOUT TST		Chi^2^	Pr	TST ≥ 5 mm		TST = 0-4 mm		Chi^2^	Pr
	n	%	n	%			n	%	n	%		
**Biological variables**												
**Sex**					12.2	< 0.001					0.1	0.747
Female	451	41.5	414	34.4			90	40.5	361	41.7		
Male	636	58.5	789	65.6			132	59.5	504	58.3		
**Age**					19.7	< 0.001					0.0	0.905
40 years or over	569	52.3	518	43.1			117	52.7	452	52.3		
Up to 39 years	518	47.7	685	56.9			105	47.3	413	47.7		
**Socio-demographic variables**												
**Place of Residence**					18.9	< 0.001					4.3	0.114
Recife	496	45.8	516	43.4			89	40.1	407	47.3		
Metropolitan Region	462	42.7	460	38.7			108	48.6	354	41.2		
Interior	124	11.5	213	17.9			25	11.3	99	11.5		
**Years of schooling**					10.4	0.001					6.2	0.013
≥ 10 years	487	44.8	459	38.2			83	37.4	404	46.7		
Up to 9 years	600	55.2	744	61.8			139	62.6	461	53.3		
**Hospital attended**					17.4	< 0.001					13.0	< 0.001
HUOC*	474	43.6	422	35.1			73	32.9	401	46.4		
HCP*	613	56.4	781	64.9			149	67.1	464	53.6		

* HCP - Correia Picanço Hospital; HUOC - Oswaldo Cruz University Hospital

**Table 2:** Distribution of habits and clinical characteristics of people living with HIV by adherence to TST and TST reactivity, Recife 2007-2010.

VARIABLES	WITH TST		WITHOUT TST		Chi^2^	Pr	TST ≥ 5 mm		TST = 0-4 mm		Chi^2^	Pr
	n	%	n	%			n	%	n	%		
**Habits**												
**Alcohol Consumption**					0.3	0.591					3.9	0.048
Non-drinker	665	61.2	666	62.3			123	55.4	542	62,7		
Drinker	422	38.8	403	37.7			99	44.6	323	37.3		
**Smoking**					2.5	0.113					7.6	0.006
No	506	46.6	519	43.2			85	39.3	421	48.7		
Yes	581	53.4	681	56.8			137	61.7	444	51.3		
**Marijuana Use**					7.6	0.006					12.8	< 0.001
No	820	75.4	842	70.3			147	66.2	673	77.8		
Yes	267	24.6	356	29.7			75	33.8	192	22.2		
**Crack use**					8.5	0.003					20.3	< 0.001
No	1030	94.80	1099	91.7			197	88.7	833	96.3		
Yes	57	5.20	100	8.3			25	11.3	32	3.7		
**Cocaine use**					7.2	0.007					5.9	0.015
No	1006	92.50	1070	89.3			197	88.7	809	96.5		
Yes	81	7.50	128	10.7			25	11.3	56	6.5		
**Clinical variables**												
**History of Contact with Tuberculosis**					1.8	0.174					2.3	0.133
No	762	70.5	873	73.1			146	66.4	616	71.5		
Yes	319	29.5	322	26.9			74	33.6	245	28.5		
**BMI**					12.7	< 0.001					0.5	0.492
Not malnourished	1010	93.1	997	88.7			209	94.1	803	92.8		
Malnourished	75	6.9	127	11.3			13	5.9	62	7.2		
**On HAART**					0.0	0.918					4.9	0.026
No	240	22.2	246	22.3			61	27.7	179	20.7		
Yes	843	77.8	855	77.7			159	72.3	684	79.3		
**Time on HAART**					38.8	< 0.001					2.8	0.096
Less than 1 year	139	12.8	202	23.3			21	9.5	118	13.6		
One year or more	948	87.2	653	76.3			201	90.5	747	86.4		
**AIDS**					24.7	< 0.001					2.9	0.087
No	189	17.9	117	10.5			47	21.9	142	16.8		
Yes	869	82.1	1002	89.5			168	78.1	701	83.2		
**CD4**					67.1	< 0.001					10.7	0.001
< 200	137	12.6	276	26.7			13	7.6	124	17.9		
≥ 200	950	87.4	758	73.3			157	92.4	570	82.1		

Tables 3 and 4 show the results of the univariate analysis of the association between biological variables (sex, age), sociodemographic factors (place of residence, years of schooling, hospital attended), life habits and clinical variables (history of contact with tuberculosis, body mass index (BMI), use of HAART, time on HAART, presence of AIDS, T-lymphocyte CD4 count) and the two outcomes studied, adherence to TST and TST reactivity.

**Table 3:** Univariate analysis of the association between biological and socio-economic variables and adherence to TST and TST reactivity, Recife 2007-2010

VARIABLES	NOT TAKING TST		TST ≥ 5 mm	
	OR (CI 95%)	P	OR (CI 95%)	P
**Biological variables**				
**Sex**				
Female	1.0	-	1.0	-
Male	1.35 (1.14-1.60)	< 0.001	1.05 (0.87-1.70)	0.250
**Age**				
40 years or over	1.0	-	1.0	-
Up to 39 years	1.45 (1.23-1.71)	< 0.001	0.98 (0.73-1.32)	0.902
**Socio-demographic variables**				
**Place of Residence**				
Recife	1.0	-	1.0	-
Metropolitan Region	0.95 (0.80-1.14)	0.63	1.40 (1.01-1.91)	0.038
Interior	1.65 (1.28-2.12)	< 0.001	1.15 (0.70-1.90)	0.569
**Years of schooling**				
≥ 10 years	1.0	-	1.0	-
Up to 9 years	1.31 (1.11-1.55)	0.001	1.47 (1.08-1.98)	0.013
**Hospital attended**				
HUOC*	1.0	-	1.0	-
HCP*	1.43 (1.21-1.70)	< 0.001	1.76 (1.29-2.40)	< 0.001

* HUOC - Oswaldo Cruz University Hospital; HCP - Correia Picanço Hospital;

**Table 4:** Univariate analysis of the association between habits and clinical variables with adherence to TST and TST reactivity, Recife 2007-2010

VARIABLES	NOT TAKING TST		TST ≥ 5 mm	
	OR (CI 95%)	P	OR (CI 95%)	P
**Habits**				
**Alcohol Consumption**				
Non-drinker	1.0	-	1.0	-
Drinker	0.95 (0.80-1.13)	0.591	1.35 (1.00-1.82)	0.048
**Smoking**				
No	1.0	-	1.0	-
Yes	1.14 (0.96-1.34)	0.113	1.53 (1.13-2.07)	0.006
**Marijuana Use**				
No	1.0	-	1.0	-
Yes	1.30 (1.08-1.56)	0.006	1.79 (1.30-2.46)	< 0.001
**Crack use**				
No	1.0	-	1.0	-
Yes	1.64 (1.17-2.30)	0.004	3.30 (1.91-5.70)	< 0.001
**Cocaine use**				
No	1.0	-	1.0	-
Yes	1.48(1.11-1.98)	0.008	1.83(1.11-3.01	< 0.001
**Clinical variables**				
**History of Contact with Tuberculosis**				
No	1.0	-	1.0	-
Yes	0.88(0.73-1.06	0.175	1.27(0.92-1.74	0.133
**BMI**				
Not malnourished	1.0	-	1.0	-
Malnourished	1.72(1.27-2.31)	< 0.001	0.80(0.92-1.75)	0.492
**On HAART**				
No	1.0	-	1.0	-
Yes	0.99(0.81-1.21)	0.918	0.68(0.48-0.95)	0.027
**Time on HAART**				
Less than 1 year	1.0	-	1.0	-
One year or more	0.47(0.37-0.60)	< 0.001	1.32(0.83-2.20)	O.221
**AIDS**				
No	1.0	-	1.0	-
Yes	1.86(1.45-2.39)	< 0.001	0.72(0.49-1.04)	0.087
**CD4**				
< 200	1.0	-	1.0	-
≥ 200	0.52(0.41-0.65)	< 0.001	2.62(1.44-4.78)	0.002

Factors associated with not taking the TST remaining in the final model of the multivariate analysis were: being male, aged up to 39 years, place of residence (living in cities in the interior of the state), being attended at the HCP, being a crack user, less time on HAART, presence of AIDS and a CD4 count ≥ 200 (Table 5).

**Table 5:** Multivariate Final Models for Not Taking TST and for TST ≥ 5 mm, RECIFE, 2007 - 2010

VARIABLES	NOT TAKING TST		TST ≥ 5 mm	
	OR (CI 95%)	p value	OR (CI 95%)	p value
**Sex**				
Female	1		-	-
Male	1.48(1.19-1.84)	< 0.001	-	-
**Age**				
40 years or over	1		-	-
Up to 39 years	1.28(1.04-1.58)	0.019	-	-
**Place of Residence**				
Recife	1		1	
Metropolitan Region	0.90(0.72-1.12)	0.357	1.67(1.15-2.42)	0.006
Interior	1.80(1.31-2.47)	< 0.001	1.00(0.54-1.87	0.975
**Hospital attended**				
HUOC*	1		1	
HCP*	1.55(1.25-1.92)	< 0.001	2.03(1.40-2.95)	< 0.001
**Crack use**				
No	1		1	
Yes	1.68(1.10-2.60)	0.020	3.48(1.76-6.90)	< 0.001
**Time on HAART**				
Less than 1 year	1		-	-
One year or more	0.65(0.50-0.85)	0.002	-	-
**AIDS**				
No	1		-	-
Yes	6.66(4.04-10.10)	< 0.001	-	-
**CD4**				
< 200	1		1	
≥ 200	0.70(0.54-0.91)	0.008	2.71(1.40-2.95)	< 0.001
**Years of schooling**				
≥ 10 years	-	-	1	
Up to 9 years	-	-	1.80(1.25-2.60)	0.002

* HUOC - Oswaldo Cruz University Hospital; HCP - Correia Picanço Hospital;

The results of the second stage of the study showed that, of the 1,087 patients who took the TST, 20.4% (222) tested positive (TST ≥ 5 mm). The mean TST was 3.9 mm (min = 0; max = 48 mm). The prevalence of TST ≥ 5 mm was 21.6% among patients with CD4 ≥ 200 and 9.49% among patients with CD4 < 200 and this difference was statistically significant (p = 0.002).

The mean CD4 count was 447.2 cells/mm3 in the group of patients who did not react to the TST and 557.9 in the group who did react and this difference was statistically significant (*p *< 0.001).

In the final multivariate model the following factors were associated with a reactivity to the TST: place of residence (living in the Metropolitan Region), being attended at the HCP, less than ten years of schooling, crack use and a CD4 count of ≥ 200 (Table 5).

## Discussion

In the present study, 52.5% of patients did not take the TST, despite the fact that it is recommended by the Brazilian Ministry of Health. The non-adherence to TST was associated with being male, being younger, living in the interior of state of Pernambuco, being attended at the HCP, being a user of crack, less time on HAART, presence of AIDS and a CD4 count < 200. Among the patients who took the TST, 20.4% (222) tested positive (TST ≥ 5 mm). TST positivity was associated with crack use, CD4 ≥ 200, being attended at the HCP, having less years of schooling and living in the Metropolitan Region of the state of Pernambuco.

Reports on TST adherence have varied from a rate of 30%, for not taking the test in a country with a low prevalence of tuberculosis [28], and a rate similar to that encountered in the present study (50.2%) from a study also carried out in Brazil [45].

It was observed that the incorporation of the TST into routine care at the two centers was not sufficient to ensure that attending physicians actually requested the test, or indeed that patients would take it, even after talks had been given to raise the awareness of professionals and patients alike on the need and pertinence of the TST for recommending LTBI treatment. This situation contradicts the interpretation of some authors that the low rate of testing is a consequence of the unavailability of TST at health care services attending patients with HIV/AIDS [4,27,41]. It is important to clarify this question, since the treatment of LTBI depends essentially on the administration of the TST [41,42,45-47], and in Brazil this is recognized as an indicator of the quality of the service [41].

A recent WHO guideline recommends that in resource-limited countries the TST might not be required for starting LTBI treatment for people living with HIV, since screening for active TB has been carried out [48]. However, the Brazilian Ministry of Health does not adopt the criterion of performing LTBI treatment for people living with HIV when a TST has not been performed or the result is unknown, based on the grounds that the benefit is greater for those with TST reactor (≥ 5 mm), as demonstrated by a systematic review [31]. The exceptions are cases of people living with HIV that relate a history of recent contact (less than two years) with TB bacillus, or those who present radiographic imaging of sequelae of pulmonary TB with no previous history of TB treatment [42,44].

The levels of adherence to TST in this study are of considerable concern, since a prevalence of TST ≥ 5 mm of 20.5% was encountered among those who took the test. This rate of reactivity must be related to the fact that patients live in a region with a high prevalence of TB. Pernambuco has an incidence coefficient of 47.6 per 100,000 inhabitants for tuberculosis and this is considered to be the highest in the Northeast region. Recife, the city where this study was developed is the Brazilian capital with the largest rates of new tuberculosis cases in the country (132 cases per 100,000 inhabitants) and the TB mortality rate was the greatest in the country, 7.7 per 100,000 inhabitants [46].

Two other studies carried out in Brazil found a prevalence of 24.8% [45] and 34.0% [17] for a positive reaction to the TST in HIV-positive individuals. At present in Brazil, around 50 million individuals are infected with *M. tuberculosis *and roughly 14% of tuberculosis cases are related to HIV [49]. This may explain the high rates for positive TST results. A lower prevalence has been reported in Atlanta (2.5%) and in Switzerland (9.4%), which lie in regions considered to have a low prevalence of tuberculosis [28,50]. Moreover, a percentage of 20.5 of TST positivity would suggest that of the 1203 individuals who did not take the TST, around 20% could also be positive. Therefore, it is possible to estimate that 245 patients lost the opportunity of being diagnosed with and treated for LTBI. Furthermore, considering that among the 222 patients with positive TST results, 21 (9.5%) have been diagnosed with active tuberculosis, it is possible to estimate that around 23 patients could have been diagnosed and treated for active TB.

The final multivariate model of factors associated with not taking the TST showed that men and younger people presented a 48% and 28% greater likelihood of not taking the test, respectively. This would mean that adherence to the TST was less frequent among those with a greater likelihood of developing of tuberculosis [30,31].

Of the life habits, only crack use remained in the final multivariate model, which is in accordance with the hypothesis that drug use impedes adherence to health care procedures. As expected, fewer people living in the interior of the state took the TST, possibly due to the distances between their homes and the health centers, which are located in the state capital, Recife. Not undergoing the TST was also associated to the diagnosis of AIDS, to less than one year on HAART and a CD4 count ≥ 200. Elzi et al. (2007) also found that the TST was performed more frequently among patients with CD4+ cell counts of < 200 cells/mL [28]. This would suggest that the more severe patients are more frequently tested for LTBI. The most aggravating factor within this group of patients is that they have a greater chance of having a non-reactive response to TST, even when present. *Mycobacterium tuberculosis *is an intracellular pathogen that principally inhabits the macrophages. Protective immunity from *M. tuberculosis *depends on the integrity of the cellular immune system. HIV infection, by damaging the immunity mediated by cells, is the most potent known risk faction for reactivation of latent TB [3]. However, it is often not possible to detect this infection using the TST and some authors consider a negative TST in an HIV-positive patient with immunodeficiency to be a false negative [51-53]. The main implication of this is that treatment of LTBI is jeopardized in patients with a non-reactive TST caused by immunodeficiency. It is thus recommended that other factors be taken into account, such as the rate of infection or disease in the community, demographic data, a history of exposure and abnormalities in the chest X-ray [42]. These factors could help to identify patients with a high risk of developing tuberculosis, for whom LTBI treatment should be considered regardless of the TST result, given that there is as yet no valid substitute for the TST in patients with HIV [54].

One striking finding of the present study is the fact that the frequency of TST was different at the two centers (p < 0.001). This suggests different levels of involvement with TB control for the two teams. In particular, it stresses the role of the attending physician in this process, as it is this member of the team who, in Brazil, decides whether to initiate treatment of LTBI or not [23], and who is thus responsible for encouraging patients to take the TST when recommended. However, attention should be drawn to the nurses' role who is generally the team member that administers the TST, and who schedules the return visit to read the test.

According to the literature, a fundamental question relating to the control of HIV/tuberculosis co-infection is a higher degree of interaction between the action taken to control the two diseases, as a way of achieving a higher level of adherence on the part of patients to screening for tuberculosis and the treatments available [55]. This should extend beyond program coordination and include point of health care delivery, where a multidisciplinary team should be present to carry out tuberculosis control activities when caring for patients with HIV/AIDS.

The involvement of a multidisciplinary team is fundamental for the adequate running of a service, with each component playing a specific role. A study conducted among patients being attended at an adult emergency unit, including those living with HIV, has shown that triage, counseling and patient education are of special importance [56]. According to this study, it was counseling that took up most of the time, reaching out not only to patients, but also to family members and partners. Tuberculosis counseling also involved the provision of information on tuberculosis and HIV, and special attention was paid to making appointments, calling patients and ensuring patients pay a return visit by giving them a free bus ticket. Chaisson and colleagues [57] have emphasized the importance of the nurses' role, noting an increase in adherence to TST from 37% to 74%, when, in addition to a food vouchers, patients were offered a consultation with a nurse. This study also emphasized the importance of having a medical assistant involved in the final interpretation and progress of each case.

The present study has certain limitations due to its design (cross-sectional), which may have caused some associations due to reverse causality. Besides this, we had to deal with the difficulties of an operational research undertaken during the routine care of patients. A selection bias can have occurred due to the difficulties involved in accessing health services. However, health care for people living with HIV in Brazil is good and in spite of working with a convenience sample of patients being attended at two separate health centers, the sample size was robust enough to detect the differences between the groups.

### Read phonetically

Furthermore, the findings of this study may contribute to a better understanding of the reasons why TB is the leading cause of death amongst people living with HIV in Brazil. Preventing TB in people living with HIV is one of the biggest challenges in countries with a reasonable prevalence of co-infected individuals.

## Conclusions

Considering that the TST is recommended by the Brazilian health authorities, the coverage for taking the test TST was low. The most serious implication of this is that the treatment for LTBI was carried out for the unidentified TST-positive patients who may consequently go on to develop TB, and eventually die. It would be of great importance to review decisions regarding the initiation of LTBI treatment being conditional on the tuberculin skin test.

## Competing interests

The authors declare that they have no competing interests.

## Authors' contributions

All authors participated in the planning and design of the research questions, the study design, data retrieval, analysis, and writing of the manuscript. All authors participated in interpreting the data and critically reviewing the manuscript. All authors read and approved the revised manuscript.

## Pre-publication history

The pre-publication history for this paper can be accessed here:

http://www.biomedcentral.com/1471-2458/11/687/prepub
